# Topological Sensitivity in the Recognition of Disoriented Figures

**DOI:** 10.1177/2041669518809717

**Published:** 2018-11-05

**Authors:** Fumio Kanbe

**Affiliations:** Faculty of Education, Hakuoh University, Oyama, Tochigi, Japan

**Keywords:** recognition of figures, topology, isomorphism, graph invariants, mental rotation

## Abstract

A previous study by the author found that discrimination latencies for figure pairs with the same topological structure (isomorphic pairs) were longer than for pairs with different topological structures (nonisomorphic pairs). These results suggest that topological sensitivity occurs during figure recognition. However, sameness was judged in terms of both shape and orientation. Using this criterion, faster discrimination of nonisomorphic pairs may have arisen from the detection of differences in the corresponding locations of the paired figures, which is not a topological property. The current study examined whether topological sensitivity occurs even when identity judgments are based on the sameness of shapes, irrespective of their orientation, where the sameness of location is not ensured. The current results suggested the involvement of topological sensitivity, indicating that processing of structural properties (invariant features) of a figure may be prioritized over processing of superficial features, such as location, length, and angles, in figure recognition.

## Introduction

How do humans recognize disoriented figures? Although some researchers have emphasized the importance of unanalytical normalization and matching processes such as mental rotation in figure recognition (Cooper, 1975; Cooper & Podgorny, 1976; Shepard & Metzler, 1971), others have suggested that the detection of orientation-independent or coordinate system-independent properties precedes normalization processes (Corballis, 1988; Corballis, Zbrodoff, Schetzer, & Butler, 1978; Eley, 1982; Takano, 1989; Treisman & Gelade, 1980).

Here, the nature of coordinate-independent properties may be better understood in terms of Klein’s hierarchy of geometry. The five types of geometry can be ordered from very specific to very general: Euclidean, similarity, affine, projective, and topological. If the properties of two figures are invariant when they undergo transformations in one geometric scheme, they are considered to be equivalent in that scheme. In Euclidean geometry, two figures are considered equivalent even if they are displaced. In similarity geometry, figures are considered equivalent even if they are uniformly expanded or contracted for orthogonal dimensions. In affine geometry, figures are equivalent even if they are uniformly expanded or contracted for orthogonal dimensions but with different rates. In projective geometry, figures are equivalent even if their parallel lines are altered in a nonparallel way. Finally, figures are topologically equivalent even if their collinearity is disrupted (Bedford, 2001). Also see Wagemans, Van Gool, Lamote, & Foster (2000).

Chen (1982) reported that a topologically different pair of figures (a solid circle vs. a hollow circle) was more accurately discriminated than topologically equivalent pairs (solid square vs. solid circle, and solid triangle vs. solid circle). Chen (1985), as well as Chen and Zhou (1997), proposed that global topological properties were detected at an early stage of figural recognition. By systematically controlling the generation of stimulus figures in experiments, Hecht and Bader (1998) found that figures with more differences in topological structure are more easily discriminated. To study binocular perception of connected four-lined figures in 3D space, Todd, Chen, and Norman (1998) altered the geometrical structure of a standard figure to make foil figures in a match-to-sample task. Their experiment included a topological condition, in which the standard and foil stimuli differed in terms of the presence or absence of an intersection of line segments, an affine condition, in which they differed in terms of the coplanarity of four lines, and a Euclidean condition, in which they differed in terms of the angle of a line that departed from a plane defined by two other lines. Participants were asked to choose the figure that had the same 3D structure as the standard. The researchers found that participant performance (i.e., latencies and error rates) was best in the topological, intermediate in the affine, and worst in the Euclidean condition.

**Figure 1.**
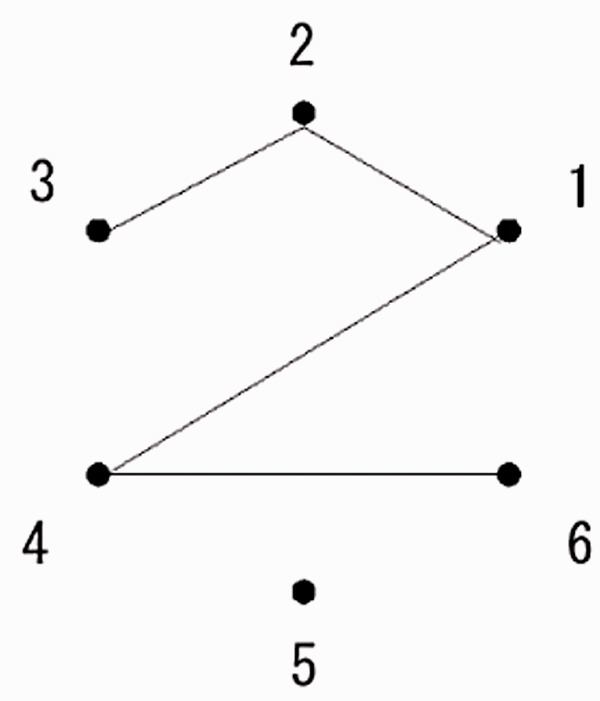
An example of a (6, 4) figure. The numbers shown near the points are labels representing the vertices of an invisible regular hexagon. The figure is specified by four pairs of point labels: 1-2, 1-4, 2-3, and 4-6.

The current study used (6 point, *n* line) figures—or (6, *n*) figures—as stimuli to investigate the recognition of planar figures. Here, a (6, *n*) figure comprises six points located at the vertices of an invisible regular hexagon and *n* line segments, respectively, connected between *n* pairs of points (Figure 1).

For figures consisting of points and line segments that span pairs of points, graph theory can provide a theoretical foundation (Harary, 1969). The application of graph theory to visual perception may be helpful for creating an accurate description of a type of stimulus consisting of points and line segments. Chen (1982) classified a solid circle, a solid triangle, and a solid square as topologically equivalent. However, in graph theory, a hollow triangle and a hollow square are not equivalent, provided that they are taken as consisting of lines spanned between vertices.

**Figure 2.**
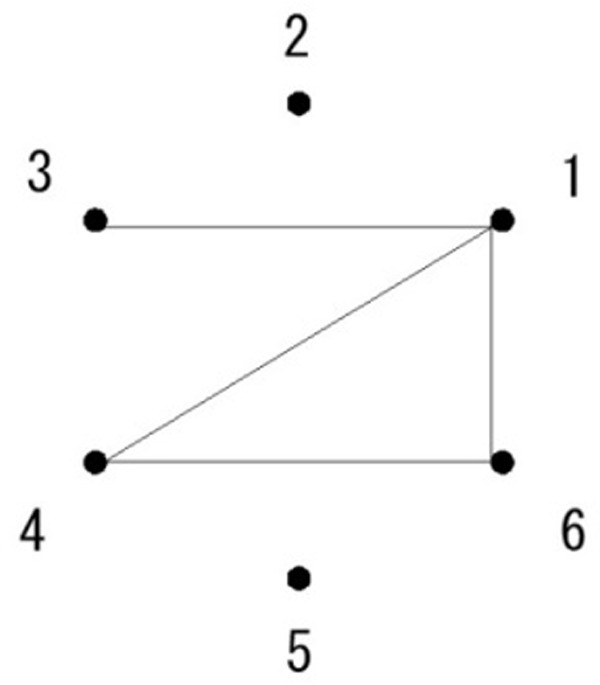
Some of the graph invariants that describe a figure. The degrees of the six points are 3 for Point 1, 0 for Point 2, 1 for Point 3, 2 for Point 4, 0 for Point 5, and 2 for Point 6. Thus, Points 2 and 5 are, respectively, isolated points, Point 3 is an endpoint, and Point 1 has the maximum number of degrees. The line segments spanning between points 1 and 4, 4 and 6, and 6 and 1 constitute a closed sequence, and thus a cycle.

In graph theory, topologically equivalent figures are considered to be mutually isomorphic. All figures belonging to the same isomorphic set share identical states of properties, termed graph invariants, and these figures are all structurally equivalent despite their different shapes. Specifically, the value of a graph invariant is the same among mutually isomorphic figures, but the location at which a graph invariant exists could vary among isomorphic figures. Examples of graph invariants include the number of line segments connecting at a specific point (or a degree of a point), the number of line terminators (or endpoints) in a figure, and the number of closed sequences of line segments (or cycles; see Figure 2). Hereafter, the term *features* is used to refer to the properties representing specific states of figures, and *invariant features* is used to refer to graph invariants. Likewise, the term *superficial features* is used to refer to the locations of invariant features, other noninvariant features, and metrics derived from locations, such as distance, length, and angle.

**Figure 3.**
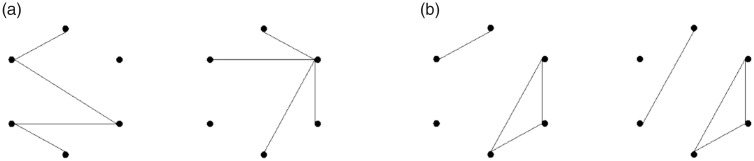
(a) A Noniso pair in which invariant feature values (e.g., maximum degrees) are different between the two figures. (b) An Iso pair in which all invariant feature values are the same but the location of an endpoint is different between the two figures.

In a series of experiments by the author (Kanbe, 2013), participants were instructed to decide whether the figures in a presented pair were the same in terms of both shape and orientation (Id pairs). As the figures in an Id pair share common invariant feature values and locations, any differences in either invariant feature values or their locations could lead to a correct *different* judgment. The results consistently showed that paired figures belonging to different isomorphic sets (Noniso pairs) were more easily discriminated than paired figures that were different in shape but belonged to the same isomorphic set (Iso pairs). These results were interpreted as an indication of the presence of sensitivity to topological differences. However, faster discrimination of Noniso pairs compared with Iso pairs may have arisen from faster detection of a difference in the locational correspondence of invariant features, rather than faster detection of differences in invariant feature values per se (Figure 3).

To clarify this issue, the present study was conducted to examine whether topological sensitivity would persist in a task in which disoriented and thus dislocated but identically shaped pairs were defined as identical. Dislocation of invariant features may not significantly affect identity decisions for paired figures. The author (Kanbe, 2015) hypothesized that figures in axisymmetric (or Ax) pairs are more difficult to discriminate compared with disoriented but identically shaped pairs because Ax pairs contain a complex shifting pattern of invariant feature locations in polar coordinates among the two figures (i.e., changes in shift direction occur about an axis of symmetry, and the distance of the shift is variable) while the figures in disoriented identical pairs have unidirectional and unisonous shifts of location (i.e., corresponding invariant feature locations have a constant angular distance). In this experiment, the shifting patterns of invariant feature locations were controlled between the two figures in each Ax pair and between those in each Iso pair. As a result, the latencies for discriminating figures were longer for Ax pairs compared with Iso pairs, even if the degree of disruption by the unisonous shifting pattern was the same. Thus, invariant feature locations do not fully explain the difficulty encountered when discriminating figures in Ax pairs.

**Figure 4.**
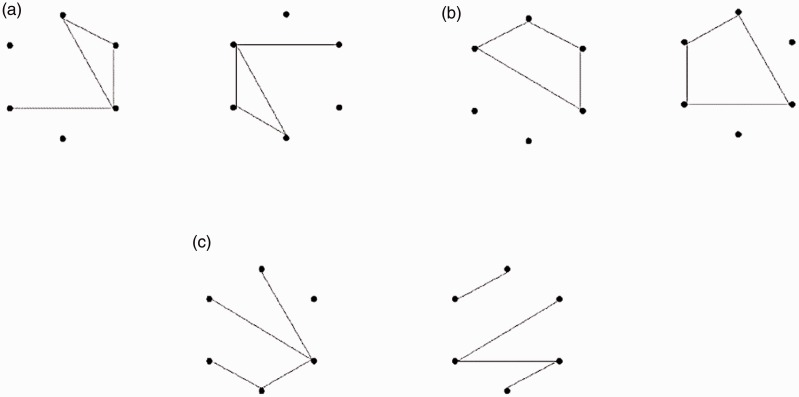
Examples of stimulus pairs presented in the experiment. (a) An Id*r* pair (*same*) with an angular distance of 180° clockwise, both figures belong to isomorphic Set 2; (b) an Iso pair (*different*), both figures belong to Set 6; and (c) a Noniso pair (*different*), left figure belongs to Set 3 and right figure to Set 4. For representative examples of isomorphic sets, see Figure 5.

In the present study, three figure pair types were randomly generated: Id*r* pairs, in which the two figures in each pair were rotated to be identical in shape; Iso pairs, in which the two figures in each pair were mutually isomorphic; and Noniso pairs, in which the two figures in each pair were not mutually isomorphic (Figure 4). Id*r* pairs contained figures in which the corresponding invariant feature values were not only identical, but their locations were constantly shifted by a specific angular distance (i.e., 0°–300° in 60° steps) from one figure to the other. As an example, Figure 4(a) demonstrates how the location of a point with a maximum degree (i.e., a point on which three line segments converge), the centroid of a cycle (i.e., a closed sequence of line segments), and the location of an endpoint (i.e., a terminating point of a line segment) are all shifted from the left figure to the right figure by 180° clockwise. As the formation of line segments in (6, *n*) figures is constrained by the locations of the six vertices (i.e., points) of an invisible regular hexagon, any superficial feature values (e.g., lengths and directions of a line segment, and the angle formed between two lines) derived from locational information are preserved after rotational transformations. For Iso pairs, all corresponding invariant feature values are the same between the two figures, but their superficial feature values are not constrained (see Figure 4(b)). For Noniso pairs, neither the invariant feature values nor the superficial feature values are constrained (see Figure 4(c)).

The occurrence of an intersection of line segments depends on the locations of the points between which line segments span, and thus, an intersection of line segments is not a graph invariant. However, Wolfe and DiMase (2003) reported that intersections are preattentively detected. Concerning differences in line length, Kanbe (2013) reported that differences in the total line lengths of pairs of figures (a superficial feature) could be confounded with topologically different pair types (i.e., simulations produced average line length differences that were smaller for Iso pairs than for Noniso pairs). Taking such potential confounds into account, only pairs of figures without intersections and the same total line lengths were used in the current study.

## Methods

This study was approved by the Hakuoh University Ethics Committee on November 19, 2012. All participants provided written informed consent prior to the start of the experiment.

### Participants

Four male and six female university students who were 19 to 22 years of age voluntarily took part in the experiment. All participants had normal or corrected-to-normal vision.

### Stimuli

Each pair of (6, 4) figures was presented on a 34 × 27 cm LCD monitor (NEC AS171MC) controlled by a NEC MJ33AA-9 microcomputer. The six vertices of each invisible regular hexagon were stylized as small filled circles with a diameter of 0.4 cm. The locations of the centers of these circles were shifted 0.2 cm outward from the vertices of the invisible regular hexagon. The shortest and longest line segments were 3.8 cm and 7.6 cm, with visual angles of 3.34° and 6.69°, respectively. Each stimulus comprised two figures that were presented simultaneously at horizontally parallel positions. The distance between the centers of the figures was 9.4 cm.

### Generation of Pairs

**Figure 5.**
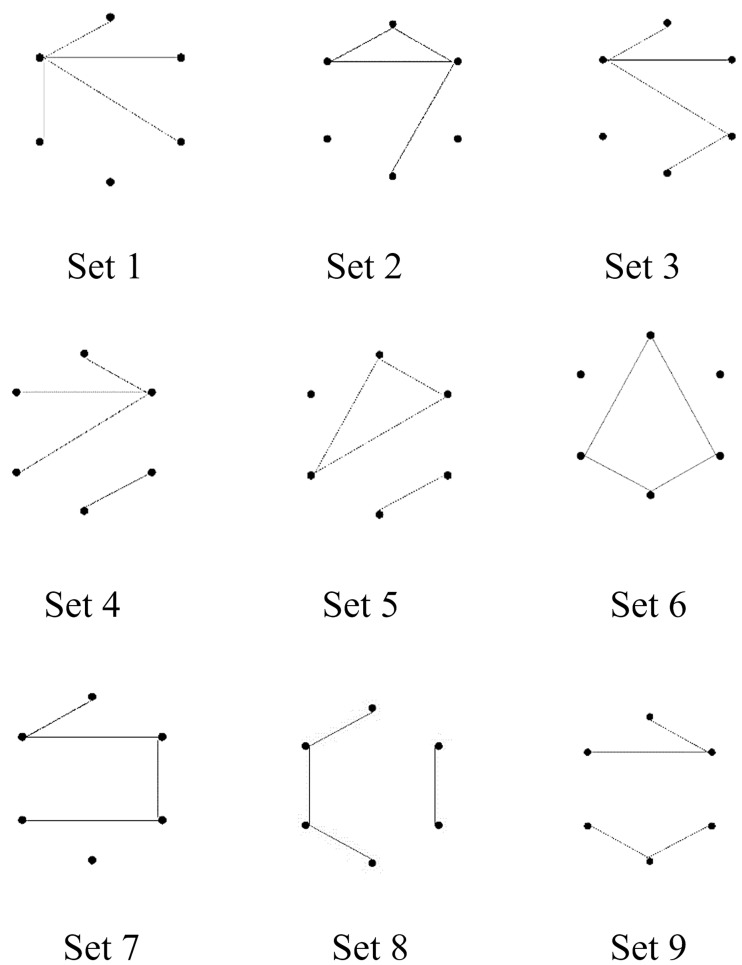
Examples of figures representing nine isomorphic sets of (6, 4) figures. The code numbers 1 to 9 designate the respective isomorphic sets and do not indicate any order.

Nine isomorphic sets of (6, 4) figures were used (Figure 5). The figures with no intersections were used as stimuli. Within each isomorphic set, two figures were randomly selected to examine whether they had the same total line length. If the figures did not have the same line length, the sampling procedure was repeated until two figures with the same total line length were selected. When two figures with the same line length were obtained, the figures were further examined to determine whether they could be rotated to be identical. If so, the two figures were classified as an Id*r* pair. If not, they were classified as an Iso pair. Within each isomorphic set, this searching process was continued until two Id*r* pairs and one Iso pair were found. This routine generated 144 Id*r* pairs and 72 Iso pairs, which were, respectively, pooled into an Id*r* set and an Iso set. To generate Noniso pairs, each isomorphic set was combined with the other set. One figure was randomly selected from one set and another figure was randomly selected from the other set to form a possible Noniso pair. If the total line lengths of the two figures were identical, the pair was pooled in a Noniso set. If not, the selection process continued until two figures with the same total line length were selected. Two selection rounds in which the exhaustive combinations of isomorphic sets were selected yielded 72 Noniso pairs. The Id*r*, Iso, and Noniso sets were then each split into two subsets comprising 72 Id*r*, 36 Iso, and 36 Noniso pairs, respectively. Next, one of the two subsets for each pair type was, respectively, concatenated into a block of 144 pairs, to be used as stimuli in a test session, and the remaining subset was concatenated into the other block of 144 pairs for the stimuli in another test session. The presentation order was randomized for both blocks of test pairs. The same sampling, concatenating, and randomization procedures were applied to generate the stimuli for two practice sessions that each included two Id*r*, one Iso, and one Noniso pair.

### Procedures

Each participant was asked to judge whether the figures in a presented pair were the *same* in shape, regardless of their orientations, or *different*. Participants indicated their decision by pushing a button on a switch box. The switch box had three horizontally aligned buttons: Enter, Left, and Right. Participants were instructed to use their index finger to push the Left button and their middle finger to push the Right button when making judgments. The set of pairs presented to each participant was divided into two blocks according to the function assigned to the Left and Right response buttons, and the functions of the buttons alternated across the blocks. The assignment of the button functions in the first block was randomized for each participant. At the start of each block, the participant received instructions regarding which response button to press to indicate that the figures were the *same* or *different*. Participants were asked to maximize speed and accuracy when responding. Each participant placed their head on a chinrest located 60 cm from the monitor. At the start of each trial, a *ready* message appeared on the screen. When a participant pushed the Enter button, the message disappeared, and a blank screen was shown for 2.5 s. Then, accompanied by a beep, a fixation cross appeared at the center of the screen for 0.5 s before it was replaced by a pair of stimulus figures. The stimulus figures remained on the screen until the participant responded. Each trial was designated as a sequence that started when the participant pushed the Enter button and ended when they made a response. A block of trials comprised four practice trials and 144 test trials. Participants received immediate feedback regarding the accuracy of each response in the practice trials, but no feedback was given in the test trials. The response latency was defined as the time that elapsed between the presentation of the stimulus and the response made by the participant.

## Results

**Figure 6.**
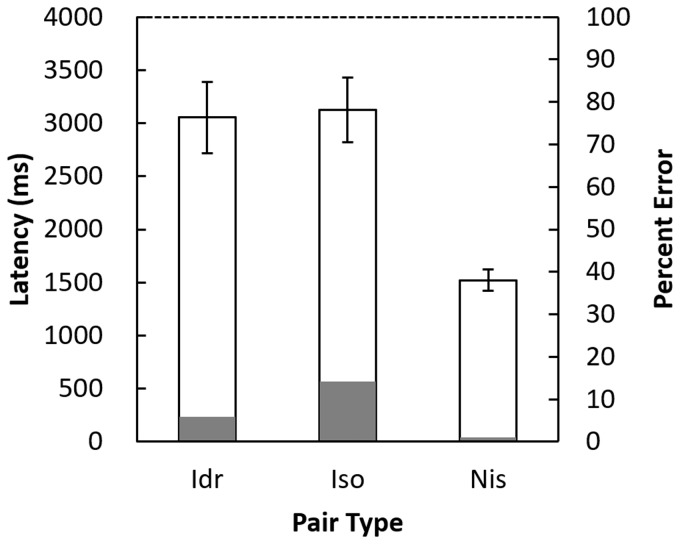
Mean latencies and *SE*s (hollow bars) and percentage errors (gray bars) of Id*r*, Iso, and Noniso (abbreviated as Nis) pairs.

The latencies and error rates of the respective pair types are shown in Figure 6. The Kruskal–Wallis test indicated that the error rates corresponding to the different pair types were significantly different, *χ*^2^(2) = 22.6, *p* < .0001. Multiple comparisons using the Steel-Dwass method revealed increasing error rates from Noniso to Id*r* to Iso pairs. The significance level of the difference between Noniso and Id*r* as well as Noniso and Iso was *p*s < .01, and that between Id*r* and Iso was *p* < .05. A repeated within-participant analysis of variance (ANOVA) revealed a significant effect of pair types on latencies, *F*(2, 18) = 42.5, *p* < .0001, *η*^2 ^= 0.45. Multiple comparisons using Scheffe’s method suggested that differences in latencies were significant between Id*r* and Noniso pairs as well as between Iso and Noniso pairs, *p*s < .01, but not between Id*r* and Iso pairs, *p* > .05. The finding that both the error rates and latencies were smallest for the Noniso pairs indicates that these pairs were easiest to discriminate. Conversely, the figures in the Iso pairs were more difficult to discern than those in the Noniso pairs.

In addition, the discriminability of figures in the isomorphic sets was also examined. A repeated within-participant ANOVA revealed a significant effect of isomorphic sets on latencies, *F*(8, 72) = 16.6, *p* < .0001, *η*^2 ^= 0.38. Multiple comparisons using Scheffe’s method suggested that the differences in latencies were significant between isomorphic Set 6 and all the other isomorphic sets, *p*s < .01, as well as between Set 5 and Set 7, *p* < .05. The differences were not significant for all the other combinations, *p*s > .05.

## Discussion

That shorter latencies were obtained for Noniso pairs compared with Iso pairs indicates that topological differences between the figures in a pair facilitate their discriminability. This supports the presence of topological sensitivity. Dislocations of invariant features between the two figures in an Id*r* pair did not affect the ease of discrimination of Noniso pairs compared with Iso pairs.

The above findings have implications for the significance of topological sensitivity in the recognition of planar figures. If a figure has no connotation of depth, as is the case with alphabetic characters, topological properties as connectivity, discreteness, and closure could be more reliable in discriminating the figure from others. Such topological properties tend to be preserved against various distortions on planar figures.

Treisman and Souther (1985) reported that a parallel search strategy is used to detect target features when the target is uniquely distinguishable, while a serial self-terminating search approach is used when a target lacks unique distinguishability. Although target features were not predefined in the current experiment, a serial self-terminating feature search and comparison model could be applied to explain the discrimination of figure pairs. That is, it is sufficient to reject identities in a Noniso pair when any difference in corresponding invariant feature values between the two figures is found. To correctly reject identities for an Iso pair, where corresponding invariant features have the same values, a value difference in superficial features (with the exception of location) must be detected. In contrast, to correctly identify an Id*r* pair, the sameness of a sufficient number of superficial features between the two figures must be confirmed, except for the locations, which must show a constant respective shift. That shorter latencies were observed for Noniso pairs compared with Iso pairs is congruent with this model in that invariant feature detection and comparisons were given priority over superficial feature detection and comparisons.

The latencies across pair types were substantially longer in this experiment compared with those in Kanbe (2013): 3,053 ms for Id*r*, 3,123 ms for Iso, and 1,522 ms for Noniso pairs in the current experiment, compared with 663 to 734 ms for Id, 656 to 725 ms for Iso, and 619 to 662 ms for Noniso pairs in Kanbe (2013). These differences are likely to have been caused by the high task demand of the current experiment (i.e., disoriented but identically shaped pairs were considered the same). The task demand not only prolonged the latencies for Id*r* pairs but also for Iso and Noniso pairs. Participants likely first attempted to detect a difference in the values of invariant features between the two figures, presumably by searching for their locations. If this were the case, invariant feature values and locations in which the invariant features exist would be treated not as discrete but as singular in the process of figural recognition.

The discrimination performance for Iso pairs in the current study may indicate that the discriminability of mutually isomorphic figures differed across isomorphic sets. Specifically, the discrimination of figures from isomorphic Set 6 was extremely easy compared with the discrimination of figure pairs in the other sets. This discrimination was even faster than the average for Noniso pairs (*M* = 1,028 ms, *SEM* = 63 ms for Set 6; *M* = 1,522 ms, *SEM* = 102 ms for Noniso overall). These findings indicate that some topologically equivalent shapes are easily discriminable. In addition, the current results suggest that certain superficial features called *shapes* can be used to effectively discriminate figures alone, although the conditions for such discrimination are likely to be limited. The figures in isomorphic Set 6 consisted of three types of quadrilaterals: rectangles, trapezoids, and elongated diamonds (see Set 6 in Figure 5). All combinations of different quadrilaterals were *different* pairs. All combinations of the same quadrilaterals with different orientations were Id*r* pairs. That is, no Ax pairs belonged to isomorphic Set 6. It has been repeatedly reported that Ax pairs are more difficult to discriminate than Id*r* pairs (Förster, Gebhardt, Lindlar, Siemann, & Delius, 1996; Kanbe, 2002, 2015). In addition, it has been proposed that closed figures (i.e., cycles) are easily recognizable (Elder & Zucker, 1993; Kanbe, 2009; Mori, 1997; Treisman & Souther, 1985). These previous findings suggest that the easy discrimination of pairs from isomorphic Set 6 in the current study may have occurred because of the absence of Ax pairs as well as the fact that the stimulus figures were all easily recognizable shapes. The properties of figures, which are intuitively interpreted as shapes, should be examined in more detail in future studies.

In conclusion, this study revealed that mutually isomorphic pairs of figures were more difficult to discriminate compared with mutually nonisomorphic pairs in a task in which participants were asked to decide whether a given pair of figures was rotated to be identical. These data support the presence of topological sensitivity, which implies that the detection and comparison of invariant features is given priority over those of superficial features in the process of figure recognition.
